# Antioxidant Effects and Mechanisms of Medicinal Plants and Their Bioactive Compounds for the Prevention and Treatment of Type 2 Diabetes: An Updated Review

**DOI:** 10.1155/2020/1356893

**Published:** 2020-02-13

**Authors:** Jeremiah Oshiomame Unuofin, Sogolo Lucky Lebelo

**Affiliations:** Department of Life and Consumer Sciences, University of South Africa, Cnr. Christiaan de Wet and Pioneer Ave., Private Bag X6, Florida 1710, South Africa

## Abstract

Diabetes mellitus is a metabolic disorder that majorly affects the endocrine gland, and it is symbolized by hyperglycemia and glucose intolerance owing to deficient insulin secretory responses and beta cell dysfunction. This ailment affects as many as 451 million people worldwide, and it is also one of the leading causes of death. In spite of the immense advances made in the development of orthodox antidiabetic drugs, these drugs are often considered not successful for the management and treatment of T2DM due to the myriad side effects associated with them. Thus, the exploration of medicinal herbs and natural products as therapeutic sources for the treatment of T2DM is promoted because they have little or no side effects. Bioactive molecules isolated from natural sources have been proven to lower blood glucose levels *via* regulating one or more of the following mechanisms: improvement of beta cell function, insulin resistance, glucose (re)absorption, and glucagon-like peptide-1 homeostasis. In recent times, the mechanisms of action of different bioactive molecules with antidiabetic properties and phytochemistry are gaining a lot of attention in the area of drug discovery. This review article presents an update of the findings from clinical research into medicinal plant therapy for T2DM.

## 1. Introduction

Diabetes mellitus is a metabolic disorder depicted by hyperglycemia (elevated levels of blood glucose) and glucose intolerance, which brings about defects of insulin secretion or insulin's action to boost glucose uptake. This disorder causes a burden worldwide because of its high rate of morbidity, mortality, and higher health costs for management and treatment. According to the International Diabetes Federation report of 2017, 451 million adults worldwide are living with diabetes, with a predicted 693 million cases by 2045 [1]. On a global level, this disorder is prevalent more in the low-income and middle-income countries with almost 50% of the cases undiagnosed. In Africa, there is a high incidence of undiagnosed diabetes cases (69.2%) with 73.7% of all deaths due to diabetes occurring before the age of 60 [1, 2], thus showing the extent to which diabetes is destroying its workforce population. In Africa and other continents of the world, type 2 diabetes accounts for over 90-95% of diabetes cases [3]. The prevalence of diabetes is rapidly increasing in South Africa with approximately 1.8 million adults suffering from diabetes mellitus (DM), while an additional 1.5 million adults remain undiagnosed [4, 5].

The economic burden of diabetes in the Republic of South Africa per person per annum was approximately R 5000 in 2010 and R 26,743.69 in 2015 [6]. This statistic only showed the cost effect of treating diabetes without addressing the cost of loss of manpower, since 60-80% of those suffering from this ailment belong to the working class and they die before the age of 60 [6]. According to the World Bank, not more than 5% of a country's gross domestic profit (GDP) should be spent on health; however, in South Africa, 8.9% of GDP is spent on health-related matters [7].

Type 2 diabetes mellitus (T2DM) is ranked among one of the most challenging global epidemics because it affects both human health and economies. The number of people plagued with T2DM worldwide in the past 20 years has more than doubled [8].

T2DM is a chronic disease caused by the complex interactions of genetic and environmental factors (dietary and lifestyle factors) [9]. The roles of both our genetic makeup and the environment are contributing factors to insulin resistance and *β*-cell dysfunction [9]. In recent times, there have been arguments saying that changes in the gene makeup cannot be the main cause for the upsurge in the prevalence of T2DM but that changes in dietary and lifestyle patterns are fundamental to grasping this epidemic [10].

The management and treatment of T2DM inflict both direct and indirect costs on the subject most especially when it is linked with other comorbidities like stroke and cancer. The global community is seriously searching for a drug which is cheap and together potent against T2DM so as to cut down the number of death cases annually [11]. Furthermore, the numerous antidiabetic therapies employed by the use of conventional drugs are laborious in the sense that most of these drugs are not a single-dose program and are most of the times taken by patients for their entire life. Also, it has been reported that adverse side effects such as diarrhoea, abdominal distention, and flatulence emanate from the intake of these drugs. Thus, these limitations have prompted the exploration of management strategies in the form of medicinal plants with antidiabetic potentials which are cost effective and have fewer side effects.

At the moment, there are a number of scientific reports on the different biological activities of phytochemicals against type 2 diabetes and diabetes. However, what is lacking is a comprehensive review that gathers experimental evidence and judiciously assesses their achievement as this would provide future research direction in the area of oxidative stress-mediated diabetes related to phytochemicals in type 2 diabetes treatment. Therefore, this review investigates the link between oxidative stress and type 2 diabetes at both the cellular and molecular levels with the aim of putting forth experimental findings on the potential of phytochemicals in type 2 diabetes treatment.

## 2. Oxidative Stress and Diabetes

Oxidative stress describes a physiological state in which the formation of reactive oxygen species (ROS) and reactive nitrogen species (RNS) attains disproportionate levels, either by excess production or reduced removal due to the overwhelming antioxidant capacity of the system [12, 13]. These highly reactive molecules are products of normal cellular metabolism, and they play crucial roles in most signalling pathways. The mitochondrion is the site where most of these highly reactive species are generated. During ATP formation in the mitochondria, electron transport and oxidative phosphorylation take place. These electrons react with oxygen (O_2_), thus forming superoxide anions (^•^O_2_^−^) which in turn reacts with molecules like Fe^2+^ and generates other reactive species (RS) such as the hydroxyl radical (^•^OH), hydrogen peroxide (H_2_O_2_), and organic peroxides [14].

Also, the production of these highly reactive molecules can be initiated in response to both extracellular and intracellular stimuli. Extracellular stimuli on plasma membrane receptors generate RS through tumor necrosis factor- (TNF-) *α*, hormones (insulin), and growth factors (platelet-derived growth factor (PDGF) and epithelial growth factor (EGF)). Intracellular stimuli that generate reactive species (RS) are induced by nicotinamide adenine dinucleotide phosphate (NADPH) oxidase [15], nitric oxide synthase (NOS) [12, 16], and mitochondrial electron transfer [17]. In addition, RS can also be generated via some enzymatic systems such as monoamine oxidase, lipoxygenase, xanthine oxidase, and glucose oxidase [15]. All of these are the major sources of reactive species (ROS and RNS), and upon their overwhelming the body system they react by modifying and damaging cellular macromolecules such as nucleic acids, proteins, lipids, and carbohydrates to generate reversible or irreversible oxidative modifications. They also have the ability to trigger a number signalling cascades linked with decoding stress, such as the mitogen-activated protein (MAP) kinase family and c-Jun *N*-terminal kinase (JNK) [18]. ROS has the ability to react with motifs of certain metal ligands such as metalloproteases and the iron in oxyhemoglobin. The superoxide radical (^•^O_2_^−^) possesses the ability to modify and inhibit catalase, while the hydroxyl radical (^•^OH), a major product of a Fenton reaction, is released during prolonged exercise and disease condition such as diabetes [19, 20].

Recent findings have revealed that ROS, most especially hydroxyl and superoxide radicals, react with certain amino acids (such as cysteine, histidine, tryptophan, methionine, and tyrosine), proteins, and simple peptides, thus making them susceptible to altered function and damage, and thus modifying their structure [20–22]. The effect of ROS and RNS on fatty acids, lipoproteins, and phospholipids induces a process called lipid peroxidation, and its resultant effect is the formation of intermediates/products such as 4-hydroxynonenal, hydroperoxide lipid, and malondialdehyde. These products cause alterations and damages to the plasma membrane, and they also have the ability to diffuse to other cells within the organism, thus causing inflammation through the binding of the oxidized low-density lipoprotein receptor and also triggering apoptosis [20, 23]. According to Tsai et al. [24] and Kawamura et al. [25], elevated blood sugar levels enhance the production of ROS during lipid degradation of low-density lipoprotein (LDL).

Hydrogen peroxide at different levels in the cell can either act as a signalling molecule that enhances cellular proliferation or prompt cell death. At low/mild concentrations, H_2_O_2_ acts as a second messenger for the triggering of NF-*κ*B and various kinases (p38 MAPK, ERK, PI3K, Akt, JAK2, and STAT), while its presence at a little higher concentration in the cell alters mitochondrial membrane integrity, thus bringing about the loss of the mitochondrial membrane potential and the release of cytochrome c and other proapoptotic proteins such as apoptosis-inducing factor (AIF) [26, 27]. Upon the liberation of cytochrome c, it triggers the activation of the intrinsic caspase-dependent apoptotic pathway [28].

Oxidative stress has been attributed to be one of the major determinants for the development of diabetes [29, 30]. The overwhelming of the antioxidant system by oxidants promotes the pathogenesis of diabetes and that is why we have more oxidative cells in diabetic subjects than in healthy subjects, i.e., a higher level of ROS production [31, 32]. Also, several reports have shown that there is a close association between oxidative stress and DM due to increased oxidative damage to vital macromolecules. According to reports of Grimsrud et al. [33] and Muellenbach et al. [34], there is an increased level of protein carbonylation and nitrosylation in insulin-sensitive tissues and in the type 2 diabetes mellitus (T2DM) state. Also, research findings have shown a strong association between increased oxidative stress and protein unfolding which causes the loss of protein function in a number of animal models [35, 36]. In diabetic patients, oxidative stress causes the alteration of two major mechanisms which are insulin resistance and insulin secretion. Oxidative stress causes the adipocytokine dysregulation and inhibition of insulin signals, thus bringing about insulin resistance. There are also increased levels of malondialdehyde (MDA), protein carbonyls, protein oxidation products, 4-hydroxy-2-nonenal, glycation end products, isoprostanes, carbohydrate modifications, and 8-hydroxy-2′-deoxyguanosine (8-OH-dG), which are biomarkers of oxidative stress in diabetic subjects [37–39]. Furthermore, the upsurge production of ROS in T2DM subjects has been shown to trigger harmful pathways such as glucosamine pathways, advanced glycation end products (AGEs), and PKC*β*1/2 kinase [40].

In addition, high levels of leptin, free fatty acids (FFA), and nonesterified FFAs promote excessive production of ROS in T2DM subjects. These unnecessary FFAs go into the tricarboxylic acid cycle to produce acetyl-CoA and loads of NADH, which causes the overproduction of mitochondrial superoxide.

## 3. The Signalling Pathways Involved in Glucose Metabolism Disorder in Diabetes

Elevated blood sugar levels have been implicated in the induction of oxidative stress *via* a number of mechanisms, viz., autoxidation of glucose, AGE formation, polyol pathway, and PKC*β*1/2 kinase [41]. Elevated free fatty acids, leptin, and other circulating factors in T2DM patients may also contribute to causing ROS overproduction [42].

**Figure 1:**
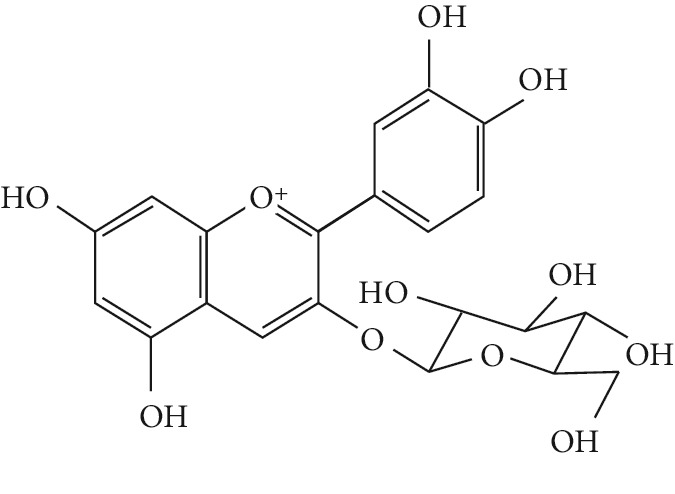
Multiple signalling pathways underlying hyperglycemic cellular damage in type 2 diabetes mellitus.

In recent years, clinical and epidemiological studies in diabetes research have confirmed that hyperglycemia and lipid metabolism abnormalities have grave influence in the onset of both micro- and macrovascular diseases. To this end, four key hypotheses have been put up through clinical trials to see specific inhibitors of hyperglycaemia causing T2DM (Figure 1). These four key hypotheses are activation of protein kinase C (PKC) isoforms, increased advanced glycation end product (AGE) formation, and increased hexosamine biosynthetic pathway flux and increased poly(ADP-ribose) pathway flux (PARP).

### 3.1. Activation of Protein Kinase C (PKC) and Diacylglycerol Formation

The protein kinase C (PKC) family consists of not less than eleven isoforms of serine-threonine kinases, which contribute to the regulation of endothelial cell permeability, stimulating cell proliferation and vascular growth [43]. According to the reports of Aiello et al. [44] and Geraldes and King [45], PKC*β* has been described to be a potential target for the improvement of diabetic complication. It was revealed that its activation is enhanced by increased glucose levels in diabetic animals and vascular cells [44, 45]. In recent times, high glucose levels induce the activation of PKC and the increase in the levels of diacylglycerol (DAG) in a number of tissues (retina, aorta, heart, and renal glomeruli) are involved in diabetic vascular complications using diabetic animal models and patients [46–48]. Also, a large amount of clinical and animal experimental models implicated elevated glucose levels to be the direct activator of the polyol pathway, and it is also linked with the excessive generation of reactive oxygen species (ROS) by the activity of mitochondria, PKC, and NADPH oxidase [49, 50]. Furthermore, prolonged activation of PKC has been linked to influencing the activation of a number of growth factors, i.e., platelet-derived growth factor (PDGF), transforming growth factor *β* (TGF*β*), and vascular endothelial growth factor (VEGF) in both cultured mesangial cells and glomeruli of diabetic rats [51, 52].

### 3.2. Increased Intracellular Formation of Advanced Glycation End Products

According to Degenhardt et al. [53], intracellular hyperglycaemia is a fundamental event in the formation of both intracellular and extracellular AGEs. Advanced glycation end products (AGEs) refer to a group of heterogeneous compounds that can arise from the intracellular autooxidation of glucose to glyoxal, the breakdown of the Amadori product (glucose-derived 1-amino-1-deoxyfructose lysine adducts) to 3-deoxyglucosone, and also the nonenzymatic removal of phosphate from glyceraldehyde phosphate and dihydroxyacetone phosphate to yield methylglyoxal [43, 53]. These reactive products (3-deoxyglucosone, glyoxal, and methylglyoxal) react with free amines of proteins and lipids and speed up development and accumulation of AGEs in the body [54, 55]. According to Piperi et al. [56], excessive production of AGEs inflicts greater injury to pancreatic beta cells than through hyperglycemia. In addition, hyperglycaemia has a direct effect on proteins of the electron transport chain by way of promoting the generation of ROS which in turn induces the fierce formation of AGEs [57, 58]. It is well known that the accumulation of AGEs is associated with the development of insulin resistance and also in the pathogenesis of diabetic complications [55, 59, 60].

According to Qiu et al. [61], extracellular AGEs aid the binding and activation of signal transduction receptor RAGE (receptor of AGE). Furthermore, the intracellular production of the AGE precursor causes damage to cells via three mechanisms: (i) modification of intracellular protein by AGEs, thereby causing the loss of function of cells; (ii) activation of the RAGE signalling axis, which results in cell apoptosis, proliferation, migration, and dysfunction; and (iii) plasma protein modification, which causes the binding of AGE precursors to AGE receptors (i.e., RAGE and AGE-R1, 2, and 3) on cells such as vascular smooth muscles and macrophages. The binding of AGE precursors to their respective receptors has been linked with a number of signalling pathway such as p21ras/ERK1/2MAPK, JAK/STAT, NADPH oxidase/ROS, and nuclear factor kappa B (NF-*κ*B) activation, therefore resulting in complications such as diabetes, cancer, aging, and neurological diseases [62].

### 3.3. Increased Polyol Pathway Flux

The reduction of a wide variety of carbonyl compounds to their respective alcohols is stimulated by a family of aldose reductase enzymes [43, 63, 64]. The poly(ADP-ribose) pathway (PARP) involves the breakdown of tissues and cells; it also consists of key enzymes such as aldose reductase (AR) and sorbitol dehydrogenase (SDH) [64–66]. During this metabolic process, glucose is reduced to its preferred corresponding alcohol-sorbitol by the action of AR instead of being phosphorylated as 6-glucose phosphate [67, 68]. These reactions make use of nicotinic acid adenine dinucleotide phosphate (NADPH). The enzyme aldo-keto reductase (AR) determines the overall rate of the polyol pathway, and it also has a low affinity (*K*_*m*_ > 100 mM) for glucose while in nondiabetic subjects, wherein the glucose concentration is normal. During the metabolism of glucose by the polyol pathway, a very minute percentage of total glucose is used [67, 69]. In a hyperglycemic state, AR activation is achieved by increased intracellular glucose. As a result of this reaction, resilient polar sorbitol is produced which struggles to seep into the cell membranes, thus bringing about osmotic cell swelling, impairment of cellular structure and function, a decrease of ATPase activity, and ultimately setting in motion cell metabolism and functional damage [43]. The oxidation of sorbitol to fructose by the action of sorbitol dehydrogenase causes PKC activation by way of an increased NADH/NAD^+^ ratio [70]. It is noteworthy that ROS is not generated in a direct way in this mechanism but it is associated with redox imbalance that brings about the onset of oxidative stress [71–73]. Recent findings have implicated PARP to be strongly associated with a myriad pathogenesis of diabetic complications, e.g., AGEs, PKC, and oxidative stress. In addition, it has been revealed to stimulate cardiac damage via its activation of NF-*κ*B (nuclear factor *κ*B) and also inducing the overexpression of vasoconstrictor endothelin-1 (ET-1) [49, 74, 75]. Furthermore, attention has shifted to PARP as one of the intense subjects in the aetiology of diabetic complications [73, 74].

### 3.4. Increased Flux through the Hexosamine Biosynthetic Pathway

Abnormally high blood sugar levels and insulin resistance-induced fatty oxidation plays a key role in the onset and advancement of diabetic complications via increasing the flux of fructose-6-phosphate into the hexosamine biosynthetic pathway [76, 77]. This abnormal blood glucose level triggers the premature activation of some metabolic pathways, which in turn causes the usual expression of certain cytokines such as CTGF, ICAM-1, PAI-1, TGF-*β*, TNF-*α*, and VCAM-1, which are involved in the development of lesion [78, 79]. Upon the absorption of glucose by cells, a majority are digested and shoved via glycogen synthesis, metabolism of the pentose phosphate, and glycolysis; furthermore, approximately 1-3% of glucose also go into the hexosamine biosynthetic pathway [79, 80]. According to Qin et al. [81], the excessive shunting of intracellular glucose via the hexosamine biosynthetic pathway has been implicated in a myriad of diabetic complications. Furthermore, the hexosamine biosynthetic pathway allows fructose 6-phosphate from glycolysis to be used as substrates for reactions that require of UDP-N-acetylglucosamine such as in the case of the formation of O-linked glycoproteins and also the synthesis of proteoglycans. Another thing peculiar to this pathway is that 6-phosphate monophosphate transaminase catalyzes its first step of reaction, and it is also the rate-limiting enzyme of the pathways [82, 83]. The ability to inhibit glutamine : fructose-6-phosphate amidotransferase (rate-limiting enzyme) which converts glucose to glucosamine helps blocks hyperglycaemia-induced increases in the transcription of TGF-*α*, TGF-*β*_1_, and PAI-1 [76, 84–86]. Lastly, the activation of the hexosamine biosynthetic pathway via hyperglycaemia could bring about the overexpression of a number of cytokines such as TGF-*α*, TGF-*β*, VEGF, and PDGF in non-insulin-sensitive tissues and also lead to the onset of diabetic complications [62].

**Figure 2:**
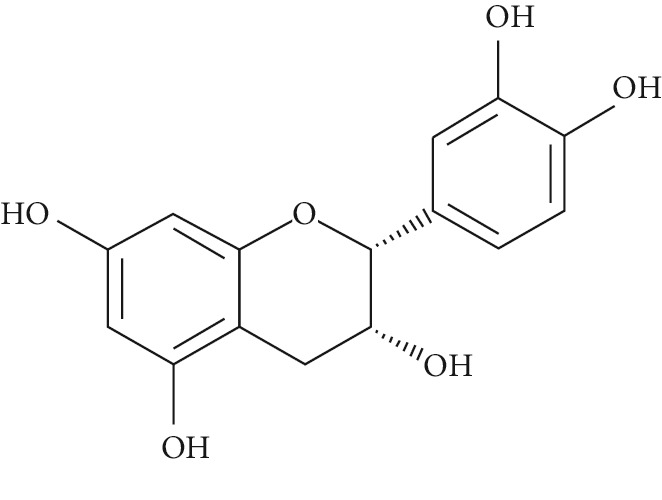
Some summarized pathways with increasing reactive oxygen species in a hyperglycemia state.

**Figure 3:**
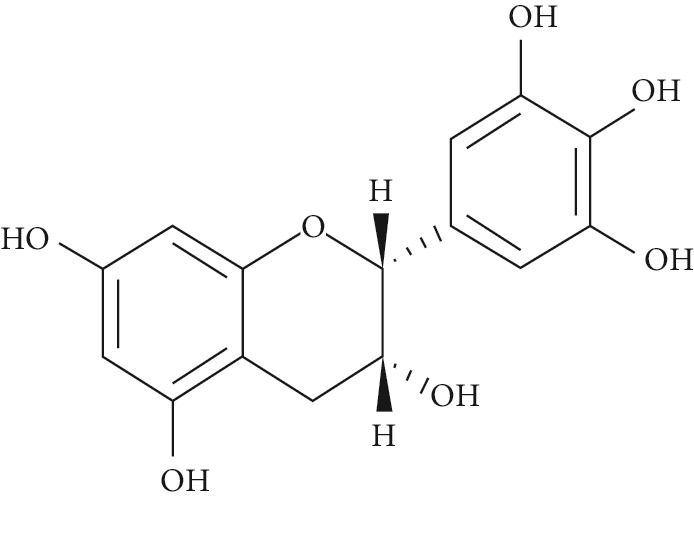
Enzymatic reactions that generate ROS in diabetic state.

Some other molecular mechanisms have also been implicated in the generation of free radicals during hyperglycemia in both *in vitro* and *in vivo* models. Such mechanisms include the mitochondrial mechanism, dysfunction of cellular antioxidative defense system (ADS), glucose autoxidation, lipid peroxidation, and activation of free-radical generator enzymes such as nicotinamide adenine dinucleotide phosphate (NADPH) oxidases, xanithine oxidase, cytochrome P450 (CYP450), myeloperoxidase, and uncoupled endothelial nitric oxide synthase (eNOS). These mechanisms are summarized in Figures 2 and 3.

### 3.5. Mitochondrial Mechanisms

The mitochondrial respiratory chain (MRC) is constituted of five multimeric enzyme complexes (I-V). The MRC is an established site for the production of free radicals throughout hyperglycemia. Research findings have highlighted the originators of mitochondria free radical formation. These include accelerated electron disposition into the electron transport chain of the mitochondria through influx electron donation aided by complexes I, III, and IV; escape of electrons; repression of the function of the mitochondria antioxidative defense system (ADS); and alteration of mitochondrial DNA [87]. The chief role of the mitochondria in the cell is energy generation (ATP) through oxidative phosphorylation. This process involves two main stages: (a) oxidation of NADH/FADH_2_ which aids in the supply of electrons to METC and (b) phosphorylation of ADP to ATP [88]. In a hyperglycemic condition, the glycolytic and tricarboxylic acid pathways cause elevated levels of NADH and FADH_2_ [76, 88], thus promoting the accumulation of electrons in complex I and ultimately aiding in the excessive production of superoxide anion (O_2_^−^) [89–91].

The escape of electrons from the mitochondria brings about free radical production via the disruption in electron transfer induced by leakages in electrons at complexes I and III and also aids the breakdown of O_2_ to ^•^O_2_^−^. When mitochondrial ADS levels are lessened, they boost free radical production. The presence of manganese-dependent superoxide dismutase (MnSOD) in the mitochondria transforms O_2_^−^ to H_2_O_2_ and O_2_. Research findings have implicated the hyperglycemic state to be one of the reasons why there is a diminished level in mitochondria ADS expression in addition to a weakened buffering potential [87, 88, 92]. Mutation in mDNA influenced by hyperglycemia promotes the decline in the level of MnSOD, peroxiredoxins (PRX), thioredoxin (TRX), and 8-hydroxydeoxyguanosine [93].

### 3.6. Dysfunction of Cellular Antioxidative Defense System

Literature is replete with information that hyperglycemia gives rise to a defect in the antioxidative defense system (ADS) [94, 95]. In a diabetic state, there are a number of decreases in some enzyme activity levels. For instance, in the brain, there is a drastic decrease in the activities of SOD, CAT, and GPx. In addition, lower levels of erythrocytes, hepatic cells, lymphocytes, and vascular endothelial cells are predominant in diabetic subjects. All these diminished levels are the resultant effects of an upsurge in the activity of free radicals [87, 96–98]. Several mechanisms have been highlighted to be the likely causes of ADS-induced diabetes. The first of such mechanisms is via insulin, which is thought to be a strong catalyst in the expression of the antioxidative enzyme system [94, 99]. Its absence/shortage could trigger an aberration in ABS expression. Antioxidative enzyme glycation and inactivation is another mode by which ADS is altered in diabetes [87, 94]. According to Kakkar et al. [100], a deficiency in insulin promotes the activation of fatty acyl-coenzyme A oxidase which results in excessive generation of H_2_O_2_. Another mode by which ADS is altered in diabetes is through several possible mechanisms that may be responsible for the effect of diabetes on the ADS. Simonyan et al. in 1987 proposed another mechanism that involves the depletion in gene expression levels of CAT and SOD by a reactive species in the event of hyperglycemia [101]. This process is aided by DNA degradation and disturbance in tRNA. Sindhu et al. [97] corroborate the findings of Simonyan et al. [101] that elevated levels of H_2_O_2_ chiefly affects DNA degradation. Finally, it has been documented that the alteration in glutathione metabolism and the decline in the activity of glutathione reductase are linked with a hyperglycemic condition.

### 3.7. Glucose Autoxidation

According to Wolff and Dean [102] and Yaribeygi et al. [87], during hyperglycemia, autoxidation of glucose takes place, and this gives rise to the generation of harmful reactive species and ketoaldehyde compounds. The production of H_2_O_2_ and malondialdehyde is linked to glucose autoxidation-induced hyperglycemia. Also, glucose autoxidation has been suggested to be the main channel for the release of reactive species in a chronic hyperglycemic state [87, 103].

### 3.8. Lipid Peroxidation

There is accumulation of harmful end products (aldehydes, alkanes, carboxylic acids, ketones, and polymerization products) during fat peroxidation in the cell membrane, and this is catalyzed by an upsurge in free radicals [87]. These harmful products elicit their deleterious effects on neighboring cells [104]. In a diabetic state, there is an increased fat peroxidation, thus promoting the production of free radicals and oxidative stress [87, 105].

### 3.9. Activation of Free-Radical Generator Enzymes

The diabetes-induced free radicals that result from enzymatic reactions and activation of the seven most important oxidative enzymes like cyclooxygenases (COX), cytochrome P450 (CYP450), lipoxygenase (LOX), myeloperoxidase (MPO), NADPH oxidase (NOX), uncoupled endothelial nitric oxide synthase (eNOS), and xanthine oxidase (XOX) contribute as much as those from the mitochondria as shown in Figure 3 [87, 88].

#### 3.9.1. Cyclooxygenase

Several studies revealed that prolonged low-grade inflammation is attendant with type 2 diabetes (T2D), and a well-defined connection with COX-mediated inflammation has been ascertained [106–108]. In the past, it was thought that COX existed in only two isoforms, i.e., COX-1 and 2 [109–111], but lately, there have been findings that corroborate the presence of COX-3 [112, 113]. COX-1 and 2 are both expressed in mammalian cells and play biological roles, while COX-3 is a splice variant of COX-1 [114, 115]. COX-1 is the most essential of all the isoforms as it is found in nearly all tissues, whereas COX-2 is conveyed in minute or trace quantities and most of the time it is released as a result of stimuli taken from mitogens, pathogens, oxidative stress, and inflammation [116–119].

According to Verma et al. [120], the COX-1 abundance level is enhanced at the initiation of diabetes and it has also been implicated with greater death rate in heart-related diseases. Guo et al. revealed that there is a vast amount of COX-2 in the vascular smooth muscle cells of the type 2 diabetes mouse model [121]; furthermore, in the coronary arterioles of diabetic subjects, there are elevated levels of COX-2 and antiapoptotic protein Bcl-2 [122, 123]. Also, the elevated levels of COX-2 inside podocytes make the kidney liable to diabetic glomerular injury which occurs by way of a (pro)renin-mediated mechanism [124]. The presence of COX-2 inhibitors in diabetic patients aid in shielding against the incidence of nephropathy [125–127]. In addition, nimesulide, a known COX-2 inhibitor, averts endothelial malfunction in the hind leg of diabetic rats [128].

#### 3.9.2. Cytochrome P450 (CYP450)

Cytochrome P450 (CYP450) is a large family of enzymes linked with drug metabolism, and they are a crucial target in drug pharmacokinetics and response. They are chiefly derived from cells of the liver but are also expressed in body tissues associated with great oxidative capability [129]. They are haemoproteins whose sole aim is to aid in the biotransformation of endogenous and exogenous compounds [129, 130]. They are mostly positioned in the sarcoplasmic reticulum and inner membrane of the mitochondria where they function in processes such as metabolism and synthesis [131]. CYP2E1 and CYP4A are the two predominant CYP450 enzymes that aid in the production of oxidants such as hydrogen peroxides, hydroxyl radicals, and anion superoxide in the body [87]. According to Bansal et al. [132], isoforms of CYP4A have the tendency for producing hydrogen peroxide and superoxide. In addition, some CYP450 isoforms (2E1, 2C6, 2C7, 3A2, 4A3, and 2A1) have been activated and implicated in the onset of hyperglycaemia via the hydroxylation of fatty acids and ketone bodies in streptozotocin-induced diabetic animals [132–134].

#### 3.9.3. Lipoxygenase

Lipoxygenases (LOXs) are a heterogeneous family that catalyzes the oxygenation of polyunsaturated fatty acids such as arachidonic acid and linoleic acid to produce their hydroperoxy derivatives and in the process generate free radicals [135–137]. The resulting ROS produced binds to the enzymes' active site while in a diabetic state they cause collateral damage to surrounding tissues upon their escape [136]. LOX enzymes and their products, such as hydroxyeicosatetraenoic acids (HETEs) and hydroxyocatadecadienoic acids, have been linked with the development of diabetes-induced oxidative stress. Hyperglycemia promotes the upregulation LOX enzymes and boost their activities [136]. An upsurge in the activities of 12/15-LOX has been associated with the pathogenesis of diabetes and atherosclerosis [138–140]. Bleich et al. revealed that 12-LOX knockout (12-LOX KO) mice were resistant to the diabetes development [141].

#### 3.9.4. Myeloperoxidase

Myeloperoxidase (MPO) belongs to a superfamily of mammalian heme peroxidase enzymes, which also includes eosinophil peroxidase (EPO) and lactoperoxidase (LPO) [142]. They possess antimicrobial and antiviral potentials because of their ability to produce ROS [143]. MPO is a protein predominantly expressed in neutrophils, while smaller expression has been observed in the monocytes and macrophages [144]. MPO utilizes H_2_O_2_ to make hypochlorous acid (HOCl) and tyrosyl free radicals which possess bactericidal potential, thus creating ROS [143, 144]. Upon the activation of neutrophils and monocytes, they employ ROS for the destruction of pathogens which acquire access into the cell; however, these radicals wield a great deal of cytotoxic effect in the host cells. In a diabetic state, MPO activation gives rise to an upsurge in the production of oxidants which exert cytotoxic and oxidative activity [87]; lingering hyperglycemia is commonly linked with elevated levels of an activated MPO enzyme [145]. Furthermore, the inhibitory effect of N-acetyl-lysyltyrosyl-cysteine amine on the MPO enzyme enhances the function of an endothelial cell and abates oxidative stress in diabetic mice [87].

#### 3.9.5. NADPH Oxidase

Nicotinamide Adenine Dinucleotide Phosphate (NADPH) oxidases (NOXs) have also been linked as one of the sources of ROS generation during a diabetic state [146]. The NOX family is composed of seven members (Nox1–Nox5, Duox1, and Duox2) that transfer electrons across the biological membranes to generate ROS and are myriads of organs in the body [147]. These different isoforms stimulate superoxide generation by causing a reduction in oxygen molecules via an electron donor (NADPH) [148]. Also, these isoforms are expressed in disparate patterns within the organs of the body. These enzymes possess diverse regulatory subunits crucial for their activity. For instance, Nox1 requires NOXO1, NOXA1, and Rac and Nox2 requires p47phox, p67phox, p40phox, and Rac, whereas NOX4 is constitutively active [149]. In addition to their various activities, Nox1 and Nox2 are renowned for their copious generation of superoxide anion as their immediate product, whereas NOX4 generates hydrogen peroxide enzymes without the slightest presence of a superoxide [150]. Fakhruddin et al. in 2017 affirmed that in a hyperglycemic state, NOX enzymes are activated directly or by way of impeding adenosine monophosphate- (AMP-) activated protein kinase [88]. Furthermore, in a hyperglycemic state, there is enhancement of NOX4 expression and oxidant production in the kidney [151], while Eid et al. [152] and Lee et al. [153] showed that in the same state, there is a subduing effect on AMP-activated protein kinase, thus bringing about the upregulation of NOX4 and ultimately promoting NOX activity in the glomerulus.

#### 3.9.6. Uncoupled Endothelial Nitric Oxide Synthase (eNOS)

Uncoupled eNOS is an occurrence symbolized by an electron transfer within the eNOS molecule by way of L-arginine oxidation, which ultimately breaks down molecular oxygen into a superoxide rather than a nitric oxide (NO) [154]. Thus, it has been revealed that uncoupled eNOS plays a dual role by way of causing an upsurge in ROS production and a decline in NO bioavailability. These two processes have been linked to the development of diabetes [155]. Xia et al. in 2017 revealed that vital physiological processes in the body (cellular proliferation, cellular signalling, platelet aggregation, and vascular tone) are dependent on NO [156]. The mechanism by which uncoupling eNOS is initiated can be grouped into four pathways, viz., accumulation of methylarginines, depletion of L-arginine, eNOS S-glutathionylation, and oxidation of tetrahydrobiopterin (BH_4_) [157–159]. Nitric oxide binds to BH_4_ as a cofactor. In a diabetic condition where BH_4_ is absent, eNOS is transformed to its monomeric form (uncoupled eNOS). In this state, eNOS enzyme basically produces O_2_^−^ instead of NO [160]. Peroxonitrite (ONOO^−^) is another powerful oxidant derived from the reaction between NO and O_2_^−^. The depleted bioaccessibility of BH_4_ in the body has been connected with diabetes development [161], and it has been suggested that it is a potential cause for endothelial dysfunction and oxidative stress in diabetes subjects [161]. Uncoupled eNOS is a major source of oxidative damage in diabetes kidneys that was reversed by BH_4_ treatment [162].

#### 3.9.7. Xanthine Oxidase

Xanthine oxidase (XO) is a metalloflavoenzyme that catalyzes the oxidation of hypoxanthine, thus causing the production of xanthine and some oxidants (e.g., superoxide and peroxynitrite) [163, 164]. XO also generates oxidants, which are key players in the T2DM development process [165–168]. In a diabetic state, there is an upsurge in XO production and the treatment with an inhibitor (allopurinol) aids in the reduction of XO activity, generation of superoxide anion, and ultimately, alleviation of oxidative stress [165]. There is an exceptionally immense upsurge in the activity of XO in a diabetic state, thus promoting oxidative damage as well as inflammatory response [169].

## 4. Transcriptional Factors and Proteins Implicated in Oxidative Stress-Mediated Diabetes

T2DM is depicted by its myriad of stimuli, decisive factors whereby proinflammatory mediators play a vital role in the onset of insulin resistance and pathogenesis of T2DM through the involvement of oxidative stress and activation of several transcriptional mediator pathways [170].

Oxidative stress has been shown to increase the production of cytokine by a number of signalling pathways. A substantial amount of research findings has revealed that oxygen derivatives act as a second messenger which activate transcription factors such as nuclear factor kappa B (NF-*κ*B), which in turn leads to the production of inflammatory cytokine such as tumor necrosis factor-*α* (TNF-*α*), interleukins (ILs), growth factors, and ECM proteins [171, 172].

### 4.1. Tumor Necrosis Factor-Alpha

The TNF superfamily contains 19 legends and 29 receptors that play a myriad of roles in the body, with all members exhibiting proinflammatory activity [173]. TNF-*α* is among the first proinflammatory biomarkers to be associated with the pathogenesis of insulin resistance and glucose-related abnormalities that link to T2DM [174, 175].

It plays a vital role in the development of insulin resistance by reducing the expression of glucose transporter type 4 (GLUT 4) that regulates insulin. It is situated in adipocytes and in skeletal and cardiac muscles [176, 177].

Recent reports have revealed the pivotal role TNF-*α* plays in the induction of tissue-specific inflammation, which brings about the pathogenesis of T2DM [178–180]. According to Swaroop et al. [181], an elevated level of TNF-*α* in the blood is associated with the development of insulin resistance and diabetes. Hu et al. [182] reported that TNF-*α* activates adhesion molecules such as intracellular adhesion molecule-1 that stimulates the growth of insulin resistance.

In addition, reports have shown that in metabolic disorders such as hyperglycemia and hyperinsulinemia, which are closely related to diabetes, there is an enhanced production of TNF-*α* from monocytes and macrophages in an *in vitro* model [183, 184]. Also, there is a positive relationship between the increase in age and levels of TNF-*α* [185].

In the pathogenesis of T2DM, increased production of TNF-*α* in adipose tissues is also related to the obesity-associated insulin resistance that leads to the development of T2DM [186]. Phytochemicals like anthocyanidins, which possess potent antioxidants, have been proven to inhibit TNF-*α* activity and its related prodiabetic effects [187, 188].

There is a cross-talk between the IKK/NF-*κ*B signalling pathway and its implicated linkage to metabolism, inflammation, and insulin action [189–191]. Almost all metabolic stress signals that are induced either intracellularly or extracellularly bring about insulin resistance or pancreatic *β*-cell dysfunction by converging on the NF-*κ*B-activating kinase IKKb. Furthermore, the IKK/NF-*κ*B pathway influences glucose metabolism via its activity on the central metabolism networks in pancreatic islets. This brings about elevated damages on the islet and also causes a malfunction in *β*-cell response to metabolic stress and proinflammatory signals in insulin-resistant subjects which are the hallmark of glucose intolerance and full-blown type 2 diabetes [190, 192, 193].

### 4.2. Transforming Growth Factor-Beta

TGF-*β* belongs to a superfamily of three isoforms. The most prevalent of this isoform is TGF-*β*_1_; it is produced in its latent form where it is intertwined with protein and concealed in the extracellular matrix. TGF-*β*_1_ is made active when its complexed form is cleaved by a proteolytic enzyme [194]. A number of research findings have pointed to a high level of TGF-*β*_1_ expression in advance glycation end products, high blood glucose level, and other outcomes of oxidative stress [195–197]. TGF-*β*_1_ has been implicated as a major stimulator of tissue fibrosis, and a prolonged dosage of TGF-*β*_1_ aids in restoring normal functioning of the kidney in type 1 and 2 diabetes experimental models [198, 199].

It is noteworthy that TGF-*β*_2_ has not been well studied in comparison with TGF-*β*_1_, but it has been associated with diabetes-related problems most especially in diabetic conditions relating to the kidney [200, 201]. In recent times, isoforms of TGF have been studied closely for its downstream effects on certain microRNA (miRNAs) species [202]. It has also been reported that extreme glucose levels may possibly upsurge transcription of TGF-*β* genes which in turn promotes the elevation levels of TGF-*β* and its downstream signalling [203–205]. Although the mode by which TGF-*β* activation causes heart problems in diabetic subjects is vague, its activation in such subjects could result from the modulation of the expression of certain changes in miRNAs. These miRNAs are noncoding ribonucleic acid molecules tasked with the responsibility of controlling the expression of genes [206]. It has been reported that miRNAs modify the focal points associated with the TGF-*β* pathway which in turn alters the signalling process of the pathway [207]. An example of such modification of miRNAs has been implicated in its ability to control ERK-MAPK activity in a diabetic state [208].

### 4.3. Plasminogen Activator Inhibitor-1

Plasminogen activator inhibitor-1 (PAI-1) is a serine protease inhibitor that functions as the principal inhibitor of tissue-type plasminogen activator and urokinase-type plasminogen activator, the activators of plasminogen and hence fibrinolysis. PAI-1 is dramatically upregulated in obesity, a complex condition associated with increased risk for myocardial infarction, accelerated atherosclerosis, hypertension, glucose intolerance, insulin resistance, hyperinsulinemia, and type 2 diabetes [209, 210]. Moreover, we recently demonstrated that PAI-1 is involved in streptozotocin-induced type 1 diabetic bone loss in female mice [211].

### 4.4. Soluble Adhesion Molecules

Diabetes and its macrovascular diabetic complications are multifactorial diseases, which could be brought about by genetic and environmental factors [212]. In most locations of diabetic macrovascular complications and hyperglycemia, there is a tendency to stimulate the initiation of inflammation in the endothelium by way of dysregulation of NOS, NF-*κ*B activation, the formation of advanced glycation end products (AGEs), and oxidative stress. Upon the activation/initiation of the endothelium in diabetic subjects, there is increased expression of soluble adhesion molecules such as E-cadherin, E-selectin, intercellular adhesion molecule 1 (ICAM-1), and vascular cell adhesion molecule 1 (VCAM-1) [213]. These aforementioned molecules enable conscription of leukocytes and also bring about their permeation into tissues at locations of macrovascular diabetic complications [214, 215]. It has been observed in both type 1 and 2 murine models that the erasure of ICAM-1 in diabetic nephropathy fends off the advancement of renal diseases [216, 217]. In addition, the impasse of ICAM-1 aids in averting blood-retinal barrier collapse and endothelial cell mutilation [218, 219].

According to Leinonen et al. [220], upon the activation of endothelial cells, some soluble adhesion molecules such as VCAM-1 and ICAM-1 are liberated which are biomarkers of the inflammatory reaction. Also, P-selectin and sICAM-1 levels are notably greater in diabetic neuropathy subjects and it has also been implicated in the weakened pace of nervous conduction [221, 222].

### 4.5. Interleukins

Type 2 diabetes mellitus (T2DM) arises out of impaired insulin secretion and insulin resistance. This metabolic disorder is connected with inflammatory responses which are typified by the modification of cytokine production such as interleukins (ILs). Interleukins have been implicated in the pathophysiology of T2DM and insulin resistance by way of their respective signalling pathways [171]. On a large scale, cytokines could either be pro- or anti-inflammatory in their activity. IL-1 has been revealed to be a key proinflammatory cytokine which is mostly liberated from immune cells, and it is concealed in certain secretory cells such as adipocytes, monocytes, macrophages, and a number of cells located around diabetic macrovascular complications [171]. IL-1 has two isoforms, IL-1*α* and *β*, with a slight difference in their biological functions. IL-1 in collaboration with other cytokines stimulate inflammation [171]. In addition, Spranger et al. [223] revealed that subjects possessing a combination of discernible IL-1*β* and uplifted IL-6 levels are three times more prone to exhibit T2D in comparison with subjects having trace IL-1*β* and dwindling IL-6 levels. Genomic analysis has pointed to certain IL-1 genes to closely link with glucose breakdown, non-insulin-dependent diabetes, and a myriad of cardiovascular diseases aftermaths [224–228].

## 5. Potentials of Phytochemicals in Type 2 Diabetes Mellitus Therapy

A number of naturally occurring chemical materials/substances known as phytochemicals (phenols, terpenoids, nitrogen-containing alkaloids, and sulphur-containing compounds) found in plants have been implicated to possess antidiabetic effects [229]. Phenolic compounds have been implicated in altering inflammatory activity (CRP, IL-6, IL-1*β*, and TNF-*α*), transpirational factor enzymes (NF-*κ*B, PPAR*γ*), and genes pertinent for the occurrence of T2DM [230].

**Figure 4:**
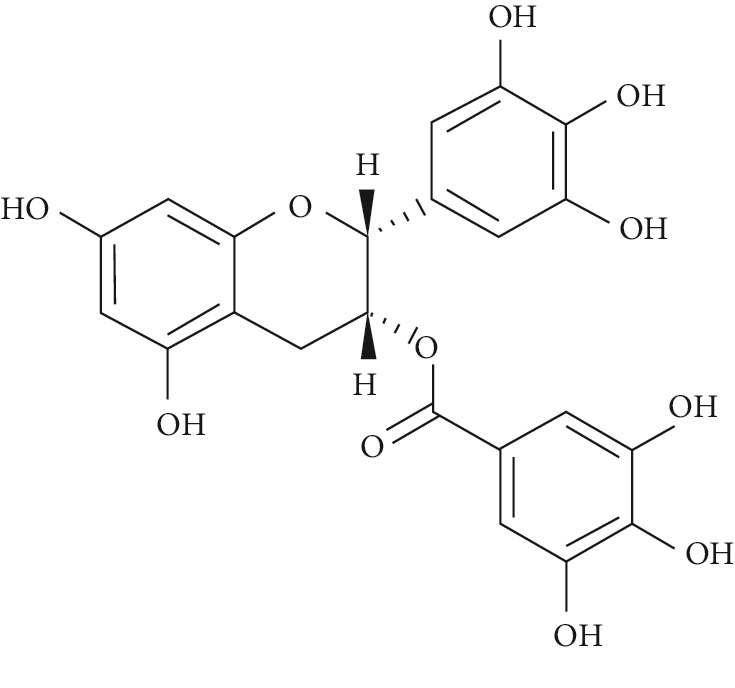
Potential targets of antioxidants in type 2 diabetes mellitus therapy.

Researchers have explored different parts of plants for their antioxidant and antidiabetic properties [231–233]. Some antioxidants present in the human body such as glutathione and thioredoxin mop up ROS via the donation of reducing equivalents in the form of a hydrogen atom or electron to the free radicals, thus making them less harmful in the body system. Certain plant-derived compounds have been ascribed with the following attributes with relation to T2DM therapy: activate the ERK1/2 and AMPK pathways [234–236]; downregulate gene expression associated with COX-2, thus promoting the increased liberation of proinflammatory mediators [237, 238]; increase glucose tolerance and insulin sensitivity [239, 240]; lessen influx of inflammatory cells [241]; decrease levels of proinflammatory cytokines IL-1*β*, IL-6, and TNF-*α* in the serum [242]; restrain the activation of NF-*κ*B pathways [243]; and repress the expression of macrophage chemostatic protein (MCP-1) and ICAM [241].  Figure 4 exemplifies the possible function of phytochemical or secondary metabolites with antioxidant potential in the oxidative stress-induced T2DM pathway. ROS/RNS influenced oxidative stress results in diabetes through the following:

1. Insulin resistance
2. Dysfunction of beta and endothelial cells due to prolonged exposure to high glucose, elevated free fatty acid level, or the combination of both
3. Decreased insulin secretion and dysfunction of mitochondrial energy product

Antioxidants embedded in natural phytocompounds have gained greater attention, and they are now being employed therapeutically for mopping up reactive species, consequently attenuating oxidative stress-mediated diabetes. Oxidative stress in a diabetic subject causes insulin resistance, beta cell dysfunction, and insulin secretion which could be modulated by phytocompounds with strong antioxidant potential via either regulating blood sugar levels or attenuating no less than one of the following mechanisms linked with insulin resistance: beta cell function, glucose (re)absorption, and incretin-related pathways [244].

## 6. Antidiabetic Effects of Phytochemicals

### 6.1. Preclinical *In Vitro*/*In Vivo* (Animal) Studies

Several plant species having hypoglycemic activity have been available in the literature; most of these plants contain bioactive compounds such as glycosides, alkaloids, terpenoids, flavonoids, carotenoids, peptidoglycans, hypoglycans, guanidine, and amino acids, that are frequently implicated as having an antidiabetic effect.

The antidiabetic property of the hydroalcoholic extract of the *Dioscorea* rhizome was revealed by its ability to reduce blood sugar level in a high-fat-induced rat model [245]. Its mode of action is its ability to attenuate insulin resistance via lessening the phosphorylation of ERK and pS6K and causing an upsurge in Akt and GLUT 4 phosphorylation [245].

Another research finding examined the antidiabetes effects and mechanism of action of *Astragalus membranaceus* root extract on a diabetic rodent model [246, 247]. The result showed that the extract has the ability to surge insulin sensitivity via Akt activation and increase receptor response to GLUT 4 [247, 248].

The ethanolic extract of *Glycyrrhiza uralensis* was able to reduce blood sugar, body fats, and blood pressure in a rat model [244, 249]. Another member of this genus, *G. foetida*, possesses a bioactive molecule (licorice) which also helps in reducing blood sugar and body fats. The mode of action of licorice is achieved by its binding and activation of PPAR*γ* which is pivotal in glucose and lipid metabolism, thus pointing to its antidiabetic potential [250].

*Gastrodia eleta* Blume is a medicinal herb in China. The aqueous extract of *G. eleta* has been shown to enhance insulin resistance by causing a reduction in body fat of diet-induced obese rats [251]. The presence of two potent bioactive molecules (vanillin and 4-hydroxybenzaldehyde) in this extract brings about its enhancement of insulin resistance by way of attenuating the fat accumulated in adipose tissues and causing a surge in fat oxidation [251].

Cinnamon (*Cinnamomum verum* and *Cinnamomum zeylanicum*) has a rich history of being used as a flavouring agent and medicinal plant for treating a myriad of ailments such as common cold, diarrhoea, diabetes, and rheumatism [252, 253]. Its antidiabetes activity is attributed to its ability to lower blood glucose levels by way of diminishing insulin resistance and promoting hepatic glycogenesis [252, 254]. Cinnamaldehyde, a water-soluble polyphenol compound isolated from cinnamon, acted as an antihyperglycemic and antihyperlipidemic agent in a diabetic rat experimental model [255].

The inclusion of *Trigonella foenum-graecum* leaves and seeds in diets of rats and dogs, respectively, revealed a significant diminishing effect on blood sugar [256]. The presence of compounds such as diosgenin, galactomannan, trigoneoside, and 4-hydroxyisoleucine in *T. foenum-graecum* promotes its antidiabetic effect [257, 258]. *T. foenum-graecum* brings to bear its hypoglycemic effect by way of enhancing/promoting insulin sensitivity in a clinical study [259].

*Semen litchi*, a common medicinal plant used by the Chinese people, also possesses antidiabetes potential. The aqueous seed extract of *S. litchi* causes a decrease in insulin resistance in a diabetic rat model [260]. In addition, a clinical study on the seeds has also corroborated its antidiabetes activity [261].

*Gymnema sylvestre* is one of the medicinal plants used in Indian folk medicine for the management and treatment of diabetes. According to Al-Romaiyan et al. [262], a novel *G*. *sylvestre* extract called OSA® showed its ability to decrease blood glucose. Its mode of action is via insulin secretion and (re)generation of beta cells in both *in vivo* and *in vitro* models. The ethanol extract of *T. divaricata* has been revealed to surge the insulin level in the blood and diminish blood sugar levels in STZ-induced diabetic mice [263]. The hydroalcoholic extract of *Carthamus tinctorius* demonstrated antidiabetic activity by way of improving insulin secretion in alloxan-treated diabetic rats [264].

*Panax ginseng* and *P. quinquefolius* are well known for their blood glucose-reducing capability in rat models [265, 266]. The ginseng mode of action of attenuating the blood glucose level is by way of diminution of beta cell function and insulin resistance [267–269]. In addition, the ethanol : water (80 : 20, *v*/*v*) extract of the ginseng root possesses a protective effect against the apoptosis of beta cells in the MIN6N8 cell line [270].

Aloeresin A, a point biomolecule derived from *Aloe vera*, has an antidiabetic potential due to an inhibitory action against alpha-glucosidase and glucose absorption in the intestine [271]. Apigenin is a flavonoid derived from Chamomile tea, which has been revealed to decrease the creation of proinflammatory cytokines such as IL-6, IL-1*β*, and TNF-*α* via modifying a myriad of signalling pathways in macrophages and as a result amending damage caused by a hyperglycemic state [272].

Baicalein is another flavonoid with antidiabetic potential isolated from the roots of *Scutelleria baicalensis* and *S. lateriflora*; its mode of action is via the activation of AMPK which results in lessened insulin resistance by way of phosphorylating AKT and insulin receptor substrate 1 (IRS-1), and inducing dephosphorylation of ERK, NF-*κ*B, and JNK [273].

Berberine is a benzylisoquinoline alkaloid derived from a majority of the *Mahonia* genus. This biomolecule has an antidiabetic property ascribed to it since it prompts a surge of insulin resistance, diminishes blood glucose levels, and accelerates beta cell rejuvenation in T2D experimental models [274–276]. Berberine also prompts a surge in glucose uptake in L6 myocytes and C2C12 skeletal muscle cell lines by way of diminution of PTP1B activity and enhancing the phosphorylation of Akt, insulin receptor, and insulin receptor substrate [277].

Curcumin, the main bioactive compound in *Curcuma longa*, possesses antioxidant, antidiabetic, and other immune-boosting effects [278]. Its antidiabetic effect is attributed to its ability to enhance beta cell function and regulate insulin tolerance [279]. According to Wongeakin et al. [280], diabetic rats fed with a dose of 300 mg/kg BW of curcumin amended vascular inflammation via attenuating ROS overproduction and ICAM-1 and NOX2 expressions.

Diosmin, is a flavonoid found in oranges, lemons, and other citrus plants. Its mode of action is by attenuating ROS-induced diabetes through the impairment of NF-*κ*B-related proinflammatory cytokines, specifically interleukins, MCP-1, and TNF-*α* [281].

Emodin, a potent bioactive compound found in *Aloe vera*, banana, and *Rheum palmatum*, has an antidiabetic property [229, 282]. Its mode of action is through the breakdown of I*κ*B, a very essential part of NF-*κ*B. In addition, the treatment of varying concentrations of emodin caused an upsurge in glucose uptake via enhancing glycogen breakdown by AMP-activated protein kinase and also aided the repression of NF-*κ*B and ERK in C2C12 myotubes and 3T3-L1 adipocytes [282].

Epigallocatechin-3-gallate (EGCG), a catechin isolated from the leaves of *Camellia sinensis* has been revealed to possess antidiabetic potential in different experimental models [283–286]. Its mechanism of action is via the upsurge of insulin secretion, safeguarding the islet of Langerhans, and diminishing both insulin tolerance and generation of glucose from FFA and lipids [286, 287].

Genistein, also known as 4′,5,7-trihydroxyisoflavone, is a naturally occurring isoflavonoid derived from *Glycine max* and some other leguminous plants like chickpeas. 4′,5,7-Trihydroxyisoflavone has the ability to sustain islet of Langerhans mass by way of upsurging the amount of beta cells and promoting its continued existence within the pancreas [288, 289]. Its antidiabetic mechanism of action is by way of initiation of ERK1/2 and protein kinase A (PKA), thus resulting in declined insulin sensitivity [290]. The treatment of genistein to high-calorie-diet mice brought about enhanced insulin action via the initiation of AMPK [291].

Kaempferol (3,4′,5,7-tetrahydroxyflavone) is a natural flavonol derived from fruits and vegetables with a very potent antioxidant activity [292]. Its antioxidant potential is due to its ability to suppress the level of IL-1*β* and TNF-*α* in diabetic neuropathy in mice [293]. Kaempferol notably reduced fast blood glucose levels of high-fat-diet mice via the initiation of the AMPK signalling pathway [294].

Morin is the main compound isolated from *Maclura pomifera*, *M. tinctoria*, and from the leaves of *Psidium guajava*. Abuohashish et al. revealed that morin diminished the surge of IL-1*β*, IL-6, and TNF-*α* through the SphK1 signalling pathway [295]. Another study using streptozotocin-induced diabetic rats showed that morin drastically trimmed down blood glucose, enzymes involved in glucose metabolism, and caused an upsurge of levels of insulin [296].

Myricetin is another naturally occurring flavonoid, but it is more abundant in walnut. A number of pharmacological properties (antioxidant, anti-inflammatory, and antidiabetic) have been ascribed to myricetin. It owes its antidiabetic effect to its ability to enhance insulin receptor substrate 1- (IRS-1-) related GLUT 4 and PI3-kinase transfer/movement [297]. According to Chang et al. [298], the effect of myricetin on HFD rats was through the improvement of PPAR*α* and the suppression of sterol regulatory element-binding protein (SREBP) hepatic expression.

Naringenin is a flavanone present in citrus and grapefruits with very strong antioxidant activity. According to Sandeep and Nandini [299], streptozotocin-induced diabetic rats treated with 0.05% of naringenin had enhanced levels of IRS 1, GLUT 1, and GLUT 3. Another study on streptozotocin-induced diabetic rats administered with naringenin displayed an improvement in the signalling pathways of both PPAR*γ* and AMPK and caused a surge in insulin sensitivity [239, 300].

Resveratrol is a stilbene abundantly found in the skin and seeds of grapes. A number of pharmacological activities such as antidiabetic, anticancer, anti-inflammatory, and immunomodulatory activities have been attributed to resveratrol [301]. The upsurge in hepatic glucose level is a crucial indicator of hyperglycemia in type 2 diabetic subjects. Resveratrol aids in the stimulation of AMPK in the liver, thus causing a decline in the production of hepatic glucose and diminishing the expression levels of certain gluconeogenic enzymes, i.e., phosphoenolpyruvate carboxykinase (PEPCK) and glucose-6-phosphatase (G6Pase) [302]. In addition, it averted apoptosis of beta cells influenced by islet amyloid polypeptide (IAPP) on culture medium [303]. Furthermore, it promotes glucose uptake in L6 myotubes by way of initiating sirtuins (SIRT1) as well as AMPK phosphorylation [304]. Clinical studies [305, 306] point toward resveratrol potential in the enhancement of glycaemic control and insulin sensitivity, and in the diminution of oxidative stress in T2DM subjects.

These selected *in vitro* and *in vivo* studies on cells and diabetic rat models directly involved or mimic cells/tissues/organs implicated in diabetes are summarized in Tables 1 and 2 thus showing the potential of phytochemicals in obtaining therapeutic agents by T2DM subjects.

### 6.2. Clinical Studies

In recent times, the use of conventional drugs for the treatment and management of diabetes has raised a lot concern from the general public because of their constitutive side effects, thus promoting the exploration of medicinal plants as alternative therapies [370, 371]. A number of medicinal plants used in the management or treatment of diabetes in folk medicine have been proven to possess a large amount of bioactive components which elicit antihyperglycemic or antidiabetic activity [371, 372]. In spite of all these great attributes and potentials ascribed to medicinal plants used for the management/treatment of diabetes in other models, there is scant information concerning their efficacy in clinical/human trials. Therefore, this section sheds light on a few medicinal plants that have been explored in clinical/human trials.

#### 6.2.1. *Allium cepa*

*Allium cepa* L. (onion) is a perennial herb in which different products (extracts, essential oil, freeze-dried powder, and juice) from bulbs have been shown to exhibit antidiabetic activity [373–378]. Eldin et al. in 2010 assessed the hypoglycaemic potential of *A. cepa* in type 1 and type 2 diabetic patients whose were administered 100 g of the crude fresh slices of *A. cepa* per day. The result revealed a significant decrease in the levels of fasting blood glucose (FBG) by about 89 mg/dL in (type 1 diabetes patients) and 40 mg/dL in (type 2 diabetes patients) after 4 hours of administration. Also, a decrease in the levels in subgroup Ib (positive control) by 145 mg/dL was observed 4 hours later [379].

#### 6.2.2. *Aloe vera*

*Aloe vera* extracts administered to three hundred and forty-eight prediabetic patients and T2DM patients for a period of 6-8 weeks revealed a substantial decrease in fasting blood glucose (FBG) [371]. It was also found that the administration of gliberclamide alone to 72 T2DM patients (49 men and 23 women) with elevated FBG levels did not ameliorate the blood glucose levels, whereas those administered 80% *Aloe vera* juice together with gliberclamide recorded a decline in the level of FBG in less than two weeks administration with no harm done in both the liver and kidney [371, 380].

In addition, *Aloe* QDM complex or placebo was orally administered during a randomized control trial (8 weeks) to one hundred and thirty-six (136) participants who were randomly assigned to the sixty-eight (68) participants from each group. The study revealed a considerable decrease in body weight, body fat mass, fasting blood glucose (FBG), fasting serum insulin (FSI), and Homeostasis Model of Assessment-Insulin Resistance (HOMA-IR) after eight weeks of treatment [381].

#### 6.2.3. Cinnamon

The antidiabetic potentials of cinnamon are highly appreciated in Ayurvedic and Chinese medicine [382] with an increase in the number of discoveries about its insulin boosting potentials [383, 384]. However, contradictory results have emerged from clinical trials using cinnamon as supplementation [371, 385, 386]. From the five clinical studies reviewed by Kirkham et al. [385], one study involves a randomized, placebo-controlled clinical trial that investigated the effect of cinnamon on blood glucose levels using a total 311 participants (eight studies) which were divided into five type 2 diabetic and three nondiabetic groups, respectively. The results revealed that only two of the diabetic groups had a significant decrease (*p* < 0.05) in FBG levels (18–29% and 10.3%), while the other three groups had no significant differences. In one of the nondiabetic groups, an 8.4% decrease was recorded (*p* < 0.01) in FBG vs. placebo when comparing with those administered with placebo, while another group recorded a significant decrease in glucose response employing oral glucose tolerance tests (*p* < 0.05). Another study by Mang et al. in 2006 examined the effects of oral administration of cinnamon extracts on 79 patients diagnosed with diabetes mellitus type 2 (T2DM) (44 men and 21 women, using oral antidiabetics or diet) using a randomized placebo-controlled clinical trial. Three grams of aqueous cinnamon extract was administered for 4 months after which a substantial decrease in fasting plasma glucose (FBG) level was recorded in the cinnamon group (10.3%) in comparison to the placebo group (3.4%); this finding corroborates the moderate hypoglycemic potential of cinnamon [387]. Gutierrez et al. in 2016 conducted a study using ten sedentary and obese females (22.7 ± 4 years; BMI 35.39 ± 5.36 kg/m^2^) who were administered 5 g of encapsulated *Cassia* cinnamon bark or 5 g of encapsulated placebo. They found out that there was a significant decrease in blood glucose levels with an improvement in glucose tolerance following OGTT by 10.1% in comparison with the placebo group [388]. However, there was no amelioration in insulin sensitivity (IS) and insulin resistance (IR) in young women. Furthermore, another double-blind, placebo-controlled study involved 72 juvenile diabetes subjects (diagnosis for ≥18 months prior to the study, age group 13–18 years) who were administered cinnamon (1 g/day) for a period of 90 days [389]. It was observed that all these studies spanned for a period of more than three months, but moderate antidiabetic effect was observed.

#### 6.2.4. *Juglans regia*

The leaves of *Juglans regia* are used in folk medicine in Iran for the treatment of diabetes mellitus and hyperglycemia, and lipid profile potential was evaluated on 61 T2DM patients [390]. Subjects used for this study were diagnosed with T2DM and had FBG values ranging between 150 and 200 mg/dL, glycated hemoglobin (HbA1c) levels between 7% and 9%, and ages between 40 and 60 years. The subjects were selected and randomly distributed into two groups: the *Juglans regia* group and the placebo group. The *J. regia* group received 100 mg capsules twice a day for a period of three months, while the control group received 100 mg placebo capsules applying the same dosage. These dosage patterns for both groups was coadministered with the standard antidiabetic therapy which is made up of metformin, glibenclamide, and nutritional regimen. The results showed that *J. regia*-treated patients had a significant reduction in the levels of FBG, HbA1c, total cholesterol, and triglyceride in comparison with the baseline and placebo group after three months of administration [390].

#### 6.2.5. *Momordica charantia*

*Momordica charantia* hypoglycemic and antihyperglycemic activities have been reported in whole plant, fruit pulp, seeds, and leaves in a number of *in vivo* studies because of the ability to reduce blood glucose levels and boost plasma insulin [391, 392].

A randomized, double-blind, active-control trial involving patients with ages between 35 and 70 years and who were recently diagnosed with type 2 diabetes (fasting plasma glucose (FPG) ≥126 mg/dL or 2 h postprandial glucose levels during 75 g oral glucose tolerance-test (OGTT) ≥200 mg/dL) was used for the study. The administration of 500 mg of dried fruit pulp (powder) which contained 0.04–0.05% charantin (2000 mg/day) to T2DM patients for a period of 4 weeks brought about a significant reduction in the levels of fructosamine without any side effects recorded [393].

#### 6.2.6. *Ocimum tenuiflorum*

*Ocimum tenuiflorum* (*Ocimum sanctum*) is popularly known as Thulasi/Tulsi in India. The ethanolic extract and fixed oil of *O. sanctum* have shown a significant antidiabetic effect *in vivo* [394, 395]. *O. tenuiflorum* is indigenous to India and certain parts of north and eastern Africa, China, Hainan Island, and Taiwan where the fresh and dried leaves are used in herbal in medicine [396].

A study conducted by Agrawal et al. in 1996 explored and studied the effects of treatment with *O. tenuiflorum* and *O. album* leaves on fasting and postprandial blood glucose and serum cholesterol levels of non-insulin-dependent diabetes mellitus patients using a randomized, placebo-controlled, crossover single blind trial. The results pointed out a substantive reduction in levels of fasting (17.6%) and postprandial blood glucose levels (7.3%) with urine glucose levels revealing a similar result [397].

Another study investigated the effect of *Ocimum sanctum* (Tulsi) on 30 young overweight/obese subjects using a randomized, parallel group, open label pilot trial. A 250 mg capsule of Tulsi extract was administered twice daily, and it brought about significant reduction in plasma insulin and insulin resistance by 28.49% and 24.79%, respectively, upon 8 weeks of administration. In addition, serum lipid level was regularized with a reduction in body weight and BMI observed when compared to the control group [398].

#### 6.2.7. *Panax ginseng*

*Panax ginseng* is a herb native to China, Japan, and Korea with distinctive branched roots [399]. The antidiabetic activity of ginseng has been documented by several authors. Kim et al. analysed data gathered from four dissimilar randomized clinical trials where the subjects were administered 0.78–6 g of ginseng per day for a period of 12 weeks. Their findings revealed that ginseng had no significantly modulated blood glucose level in T2DM patients [400]. On the contrary, findings from Shergis et al. in 2013 showed some encouragement as ginseng boosted glucose metabolism in a survey of six clinical trials [401]. In addition, another study by Shishtar et al. assessed sixteen trials where subjects with and without diabetes were subjected to the intake of different ginseng preparations (0.1–20 g/day) for a period of 4–24 weeks. The findings showed that both groups (diabetic and nondiabetic) had a significant decrease in fasting blood sugar levels [402].

#### 6.2.8. *Sauropus androgynus*

*Sauropus androgynus* is one of the most popular herbs in Asia because of its slimming potential, and it has also been shown to possess an antidiabetic potential [403]. Its antidiabetic potential was validated in a clinical trial using 18 type 1 diabetes mellitus subjects between 50 and 65 years and having body weights of 70-85 kg. The results revealed a substantive decline in blood glucose levels as reflected in glycemic index (GI) scores (GI = 55) which were far less than the glucose level in the control group (GI = 100) [404].

#### 6.2.9. *Vitis vinifera*

Hokayem et al. in 2013 investigated the clinical efficacy of nutritional doses of grape polyphenols (PPs) capable of abating fructose-induced oxidative stress and insulin resistance in thirty-eight (38) healthy overweight/obese first-degree relatives of T2DM patients (18 men and 20 women, aged 30–65 years, BMI between 25 and 35 kg/m^2^, waist circumference > 94 cm for men and >80 cm for women, FBG < 110 mg/dL) using a randomized, double-blind controlled trial between a grape PP (2 g/day) group and a placebo (PCB) group [405].

After 8 and 9 weeks of supplementation, the mixture of grape PPs at tested nutritional doses abated fructose-induced oxidative stress and insulin resistance, thus making it a vital agent in the area of preventive nutrition [405].

A new insight into the field of phytochemicals in diabetes mellitus is in their ability to act as a subordinate for allopathic drugs used for diabetes management and treatment due to their antioxidant potential which helps in combating oxidative stress and other cellular damages or side effects associated with the intake of allopathic medicine during diabetes treatment [406]. It is also worth mentioning that vitamin supplementation in T2DM subjects aids in improving the antioxidant status through a lot of ways such as causing a surge in the levels of glutathione peroxidase (GPx), superoxide dismutase enzyme (SOD), and total antioxidant capacity (TAC) and also mitigating malondialdehyde (MDA) and thiobarbituric acid reactive substance (TBARS) products. Results obtained from Balbi et al. (2018) revealed that postdiagnosis supplementation of vitamin E (alone or in combination) in T2DM promoted health benefits such as enhancement of plasma antioxidant capacity and the concentration of enzymes (GPx and SOD) and lower levels of MDA and TBARS products [406]. Another study revealed that vitamins C and E are the most frequently administered antioxidants used as supplements in subjects with type 2 DM. They also noted that the supplementation period was between 3 and 12 weeks [407]. T2DM patients are more susceptible to micro- and macrovascular complications; thus, daily intake of vitamins as supplements offers a new approach for metabolic control, as well as summing up the effect of diet, exercise, and medication.

## 7. Conclusion

Reactive oxygen species produced either exogenously or endogenously are one of major contributors to the development/initiation of T2DM, an ailment that is causing increasing morbidity and mortality in humans all over the globe. Researchers in different parts of the world are exploring natural sources (medicinal plants and natural products) for the management and treatment of diabetes mellitus because the available allopathic drugs sold over the counter are very expensive and come with a number of worrisome side effects. As a result, the exploration of medicinal plants with antidiabetic potential is gaining greater attention on a daily basis because of the presence of potent phytochemicals. In recent times, research findings have revealed that these plant chemicals possess the ability of mitigating diabetes mellitus via a number of mechanisms such as the regulation of insulin signalling, which induces gene and protein expression; the promotion of insulin secretion; the improvement of *β*-cell function; and the (re)absorption of glucose in both *in vitro* and *in vivo* models. However, only a few of these active compounds from natural sources have been translated to clinical use. An example of such is metformin derived from *Galega officinalis*. Hence, there is a need for more scientific progress in the area of converting phytochemicals with antidiabetes activity to clinical drugs as a means of reforming the management/treatment of T2DM in years to come.

## Figures and Tables

**Table 1:** Plant extracts that elicited their antidiabetic potential using both alloxan and streptozotocin-induced diabetic rats.

Number	Plant name	Plant part used	Extract used	Mechanism of action	Experiment model	References
1	*Acacia arabica*	Bark	Chloroform	Cut down the level of serum glucose and ameliorate total cholesterol (TC), triglyceride (TG), and high-density lipoprotein (HDL) and low-density lipoprotein (LDL) levels	Alloxan-induced diabetic albino rats	[307]
				Cut down levels of serum glucose, TC, TG, LDL, and malondialdehyde (MDA) levels and also boost HDL and coenzyme Q10	Streptozotocin-induced diabetic rats	[308]

2	*Acacia nilotica*	Pods	Alcoholic	Aids in depleting levels of blood glucose Boosts antioxidant enzyme system (SOD and GSH), NO level, and LPO of the kidney	Streptozotocin-induced diabetic rats	[309]

3	*Achyranthes rubrofusca*	Leaves	Aqueous and ethanolic	Reduction in levels of blood glucose and also aids in boosting levels of superoxide dismutase (SOD), catalase (CAT), and glutathione levels	Alloxan-induced diabetic albino rats	[310]

4	*Albizzia lebbeck* Benth	Stem bark	Methanol and dichloromethane	Cutting down levels of fasting blood glucose (FBG) and glycated hemoglobin and ameliorating plasma insulin. Furthermore, it caused significant diminution in levels of TC, TG, LDL, and VLDL while causing an upsurge in the level of HDL.	Streptozotocin-induced diabetic rats	[311]
		Stem	Methanolic	Reducing levels of serum glucose, creatinine, urea, TC, TG, LDL, and VLDL and on the other hand boosting HDL level	Streptozotocin-nicotinamide-induced diabetic rats	[312]

5	*Aloe vera*	Leaves	Aqueous	Cutting down the levels of blood glucose, TG, LDL, and TC	Streptozotocin-induced diabetic mice	[313]
				Ameliorating insulin secretion and pancreatic *β*-cell function, i.e., boosting pancreatic islet mass	Streptozotocin-induced diabetic rats	[314]
				Ameliorating glucose metabolism by way of cutting down the level of blood glucose	Alloxan-induced diabetic rats	[315]

6	*Artemisia afra*	Leaves	Aqueous	Rejuvenate pancreatic beta cells Inspire insulin release and ameliorate oxidative stress in the pancreas Boost glucose utilization	Streptozotocin-induced diabetic rats	[316]

7	*Barleria prionitis*	Leaves and root	Alcoholic	Cutting down levels of blood glucose and glycosylated hemoglobin Boosting levels of serum insulin and liver glycogen	Alloxan-induced diabetic rats	[317]

8	*Boerhaavia diffusa*	Leaves	Aqueous	Stimulates glucose utilization and bolsters ionic balance, renal Na^+^-K^+^ ATPase activity, and renal antioxidant status (GPx, catalase, SOD, and GSH)	Streptozotocin-induced diabetic rats	[318]
				Upsurge in levels of hepatic glucose-6-phosphatase and fructose-1,6-bisphosphatase	Alloxan-induced diabetic rats	[319]

9	*Bougainvillea spectabilis*	Roots and barks	Aqueous and methanolic	Improve the activity of glucose-6-phosphate dehydrogenase and hepatic, skeletal muscle glycogen	Streptozotocin-induced diabetic rats	[320]
			3-O-methyl-chiroinositol, a bioactive compound isolated from *B. spectabilis*	Rejuvenate pancreatic beta cells, thus causing a rise in plasma insulin and c-peptide Employ insulin-like effects	Streptozotocin- and alloxan-induced diabetic rats	[321].

10	*Byrsonima crassifolia*	Fruits and seeds	Hexane and chloroform	Upsurge in levels of CAT, GSH, GSSG, and SOD Boosting activities of glucose-6-phosphatase (G6Pase), hepatic glycogen content, and plasma insulin and cutting down blood glucose level	Streptozotocin-induced diabetic rats	[322]

11	*Caesalpinia ferrea*	Stem bark	Aqueous	Cutting down the level of blood glucose, TC, and TG	Streptozotocin-induced diabetic rats	[323]

12	*Casearia esculenta*	Root	Aqueous	Rejuvenation in levels of glucose, urea, uric acid, creatinine, and albumin; the albumin/globulin ratio; and marker enzymes AST, ALT, alkaline phosphatase (ALP), and *γ*-glutamyltranspeptidase (GGT)	Streptozotocin-induced diabetic rats	[324]
13	*Cassia fistula*	Stem bark	Alcoholic	Cutting down the level of blood glucose and also rejuvenating the levels of serum cholesterol, TG, creatinine, albumin, total proteins, and body weight	Alloxan-induced diabetic rats	[325]

14	*Catharanthus roseus*	Leaves and twigs	Dichloromethane-methanol	Cut down levels of blood glucose and hepatic enzyme activities such as glycogen synthase, glucose 6-phosphate dehydrogenase, succinate dehydrogenase, and malate dehydrogenase	Streptozotocin-induced diabetic rats	[326]

15	*Ceriops decandra*	Leaves	Ethanol	Adjusting levels of blood glucose, hemoglobin, liver glycogen, and some carbohydrate metabolic enzymes in comparison with the control group	Normal and alloxan-induced diabetic rats	[327]

16	*Cinnamomum zeylanicum*	Hydroalcohol	Whole plant (cinnamon polyphenols)	Diminish the expressions of inducible nitric oxide synthase (iNOS) and nuclear transcription factor-*κ*B (NF-*κ*B) and also rejuvenate pancreatic beta cells	Streptozotocin-induced diabetes	[255]

17	*Cistus laurifolius*	Leaves	Ethanol Three known flavonoids (quercetin-3-O-methyl ether, quercetin, and genkwanin)	Cut down the level of blood glucose level and inhibit activities of *α*-amylase and *α*-glucosidase	Streptozotocin-induced diabetic rats	[328]

18	*Citrullus colocynthis*	Roots	Aqueous, chloroform, and ethanol	Cut down the levels of blood glucose in comparison with the control group	Normal and alloxan-induced diabetic rats	[329]

19	*Combretum lanceolatum*	Flowers	Ethanol	Activation of AMPK in the liver and also inhibiting hepatic glucose production	Streptozotocin-induced diabetic rats	[330]

20	*Emblica officinalis*	Fruits, leaves	Hydromethanol	(1) Boost high-density lipoprotein-cholesterol level and diminish low-density lipoprotein-cholesterol level (2) Upsurge in the levels of GSH, GPx, SOD, and CAT (3) Boost activities of hepatic and renal SOD and CAT (4) Cut down the level of thiobarbituric acid reactive substances (TBARS)	Streptozotocin-induced diabetic rats	[331]

21	*Gynura procumbens*	Leaves	Aqueous	Enhancement of glucose uptake in the muscles	Streptozotocin-induced diabetic rats	[332]

22	*Helicteres isora*	Roots	Butanol and aqueous ethanol	Cut down levels of blood glucose, TC, TG, and urea levels	Alloxan-induced diabetic rats	[333]

23	*Hiptage benghalensis*	Leaves	Methanolic	Rejuvenation of pancreatic beta cells and improvement of insulin secretion, thus bringing about a reduction in the level of blood glucose	Alloxan-induced diabetic rats	[334]

24	*Hyptis suaveolens*	Leaves	50% aqueous ethanol	Induction of glucose utilization	Streptozotocin-induced rats	[335]

25	*Kigelia pinnata*	Flowers	Methanol	Cuts down levels of blood glucose, serum cholesterol, and triglycerides	Streptozotocin-induced diabetic rats	[336]

26	*Momordica charantia*	Seeds	Methanol	Cutting down levels of serum glucose, insulin, TNF-*α*, and interleukin 6 (IL-6)	Streptozotocin-induced diabetic rat	[337]

27	*Murraya koenigii*	Leaves	Aqueous	Cuts down level of blood glucose	Alloxan-induced diabetic rats	[338]

28	*Parquetina nigrescens*	Leaves	Aqueous	Cuts down level of blood glucose via boosting the level insulin and reducing lipogenesis	Alloxan-induced diabetic rats	[339]

29	*Phoenix dactylifera*	Leaves	70% ethanol	Cuts down levels of blood glucose, TC, and TG levels and also causes an upsurge in the level of insulin when compared with the control group	Alloxan-induced diabetic rats	[340]

30	*Phyllanthus niruri*	Aerial parts	Methanol	Cuts down levels of blood glucose, TC, and TG levels	Alloxan-induced diabetic rats	[341]

31	*Pongamia pinnata*	Leaves	Petroleum ether, chloroform, ethanol, and water	Reduction in the level of blood glucose level	Streptozotocin- and alloxan-induced diabetic rats	[342, 343]

32	*Psidium guajava*	Fruits	Aqueous	Cuts down the levels of blood glucose and lipid profile Rejuvenate pancreatic *β*-cells, thus boosting insulin secretion Suppresses pancreatic nuclear factor kappa B expression	Streptozotocin-induced diabetic rat	[344]

33	*Sida cordifolia*	Aerial parts	Methanol and aqueous	Cut down levels of serum glucose level, insulin, and cholesterol	Streptozotocin-induced diabetic rats	[345]

33	*Sphaeranthus indicus*	Roots and stolons	Ethanol	Cut down levels of blood glucose and boost levels of hepatic glycogen and plasma insulin	Streptozotocin-nicotinamide diabetic rats	[346]

34	*Terminalia bellerica*	Fruits	Methanol	Enhance insulin secretion via modulating levels of cAMP and intracellular calcium in the pancreatic *β*-cells	Streptozotocin-induced diabetic rats	[347]

35	*Terminalia chebula*	Seeds	Chloroform	Cuts down levels of blood glucose	Streptozotocin-induced diabetic rats	[348]

36	*Trigonella foenum-graecum*	Seeds	Ethanol	Cuts down levels of blood glucose	Alloxan-induced diabetic rats	[349]

37	*Zaleya decandra*	Roots	Ethanol	Cuts down levels of blood glucose, TC, TG, total proteins, urea, creatinine, and lipid peroxidation	Alloxan-induced diabetic rats	[350]

**Table 2:** Plant sources, structures, and antidiabetic mechanisms of some potential antidiabetic phytochemicals on different cell lines.

Number	Plant	Phytochemical isolated	Assay used	Antidiabetic mechanism	Structure	Reference
1	Black beans	Cyanidin 3-Glucoside	Adipocyte 3T3-L1	Upsurge in adipocyte glucose uptake, improvement in GLUT 4 expression and translocation, elevation in nuclear PPAR*γ* activity, improvement in insulin resistance	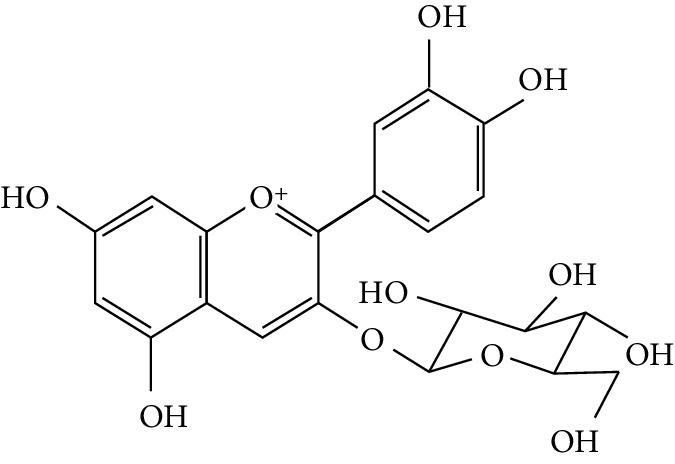	[351, 352]

2	*Camellia sinensis* (green tea), *Acacia karroo*, *Harungana madagascariensis*, and *Prunus africana*	(–)-Epicatechin (EP)	3T3-L1 adipocytes	Promote the translocation of GLUT 4 through the activation of PI3K and elevation in the phosphorylation of PKC*λ*/*ζ*	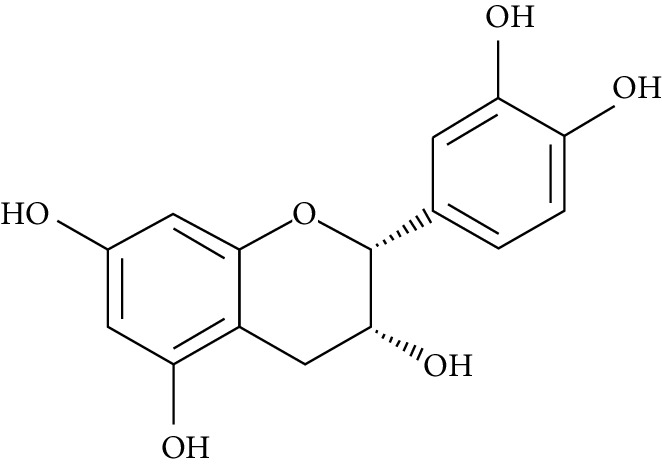	[353]
		(-)-Epigallocatechin (EGC)	3T3-L1 adipocytes	Upgrade the translocation of GLUT 4 by way of stimulation of PI3K and elevation in the phosphorylation of PKC*λ*/*ζ*	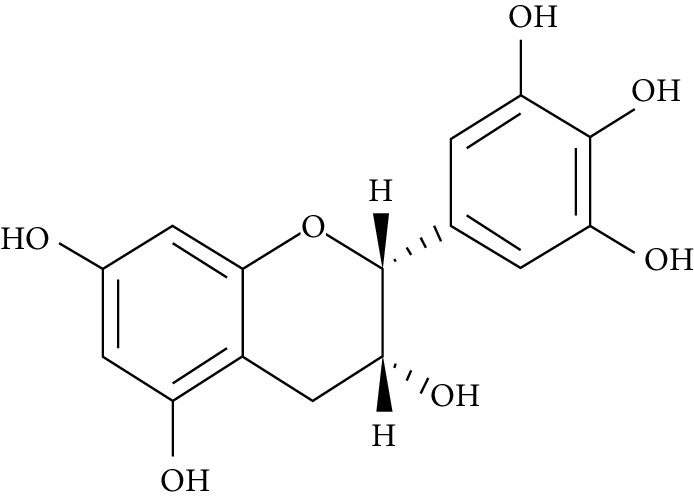	[353]

3		(–)-Epigallocatechin-3-gallate (EGCG)	3T3-L1 adipocytes	Weakens JNK phosphorylation and elevates GLUT 4 translocation	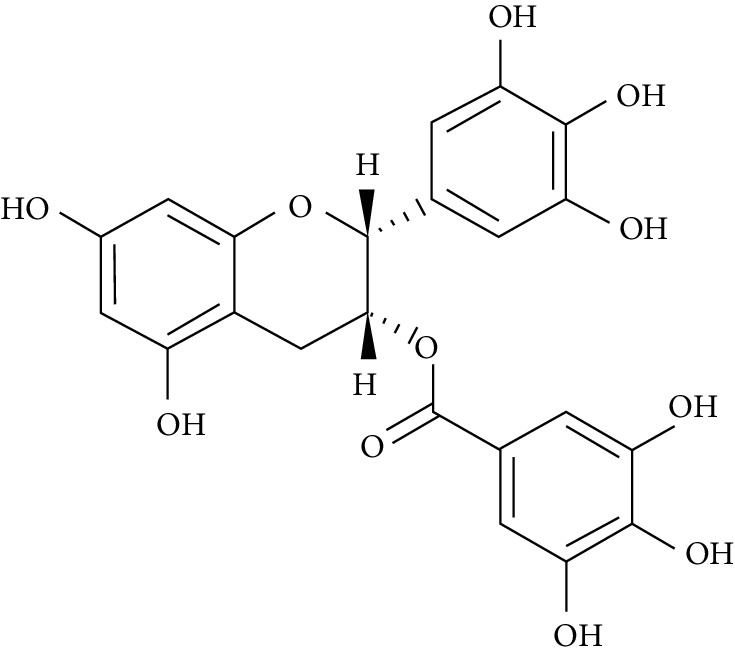	[354–359]
			H4IIE cells	Accelerates the PI3K/aPKC*λ*, AMPK, and NF-*κ*B pathways		
			Insulin-resistant L6 myotubes	Quickens glucose uptake and accelerates translocation of GLUT 4 to plasma membrane in skeletal muscle		
			HepG2	Mitigates insulin signalling blockade by reducing IRS-1 Ser307 phosphorylation through the AMPK activation pathway		
			L6 cells	Enhances glucose uptake by expanding GLUT 4 translocation to plasma membrane		
			L6E9 myotubes and 3T3-L1 adipocytes	Accelerates glucose uptake as well as GLUT 4 expression and translocation		

4	*Catharanthus roseus*, *Acalypha wilkesiana*, and *Elaeodendron croceum*	Naringenin	L6 myotubes	Accelerated glucose uptake and enhanced AMPK activation	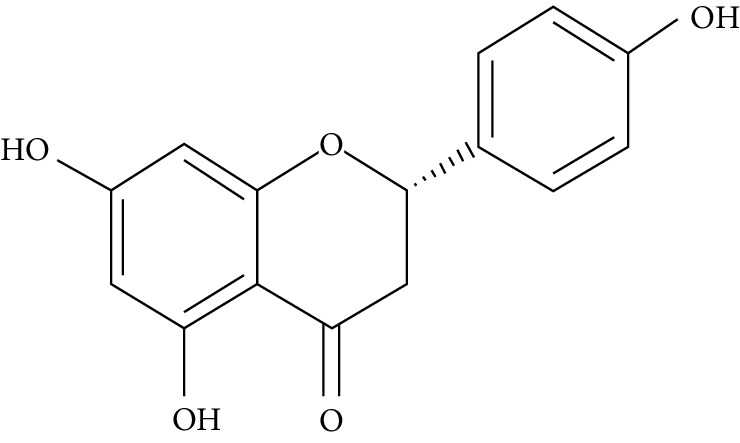	[360]

5	*Drynaria fortunei* (Kunze) J. Sm., *Citrus aurantium* L., and *Citrus medica* L.	Naringin	L6 myotubes	Accelerated glucose uptake and enhanced AMPK activation	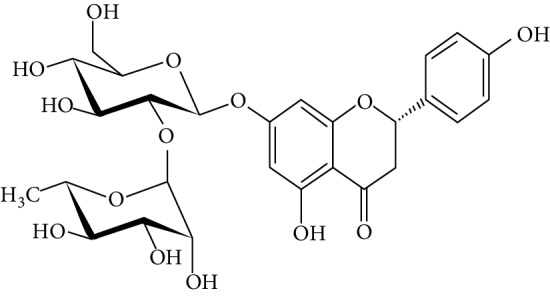	[360]

6	*Averrhoa carambola* L	Apigenin-6-C-*β*-L-fucopyranoside	Rat soleus muscle	Acceleration of insulin secretion and glycogen synthesis and cutting down blood glucose level	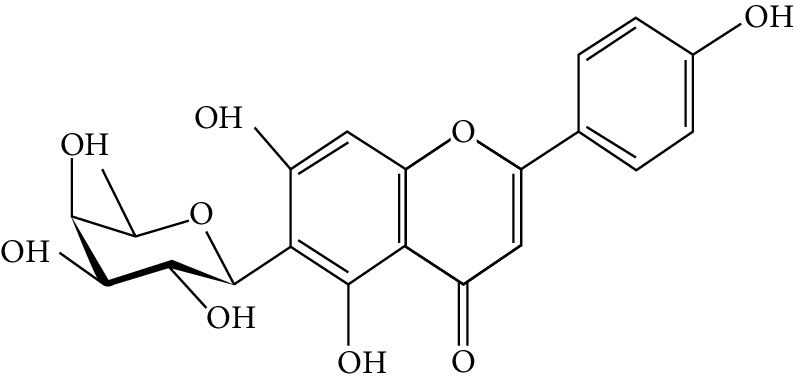	[361]

7	*Citrus tangerine*, *Citrus reticulata*, and *Citrus depressa*	Tangeritin	C2C12 myotubes	Phosphorylated AMPK and AS160 and enhanced glucose uptake and GLUT 4 translocation	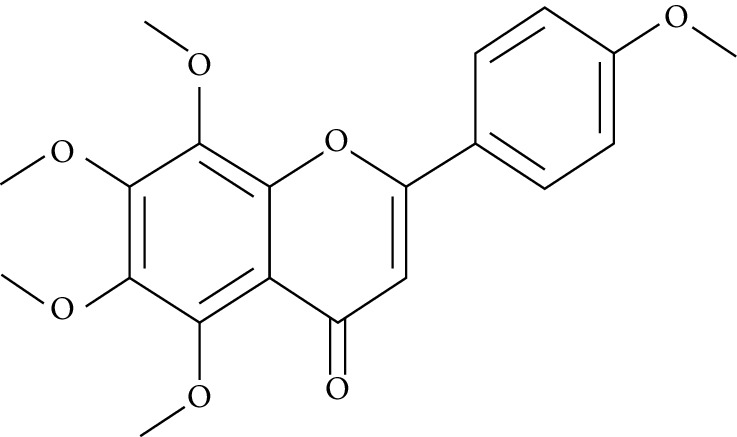	[362, 363]
			3T3-F442A adipocytes	Accelerated glucose uptake		

8	*Justicia spicigera*	Kaempferitrin	Rat soleus muscle	Acceleration of glucose uptake, GLUT 4 translocation, and glucose homeostasis	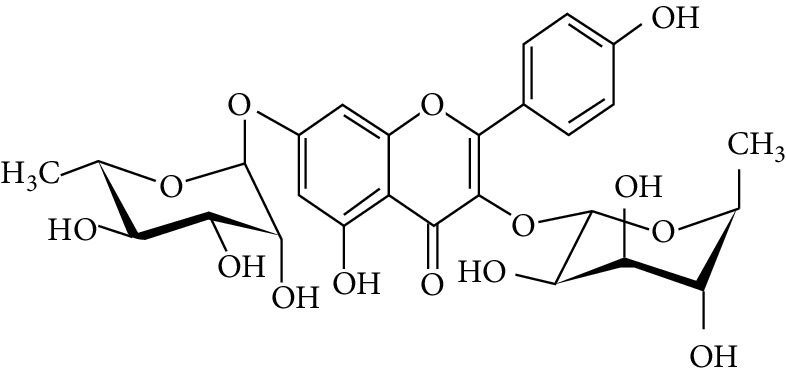	[364]

9	*Euonymus alatus*	Kaempferol	3T3-L1 adipocytes	Enhanced glucose uptake and mitigated hyperglycemia and PPAR*γ* agonist activity	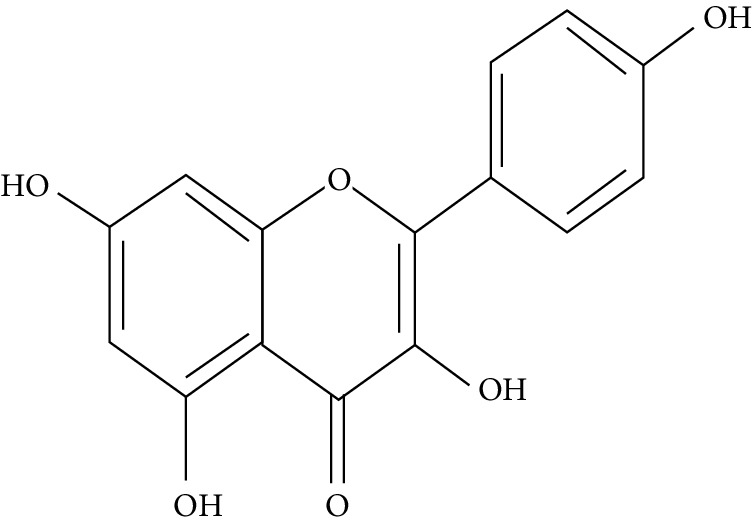	[365]

10	*Beaumontia grandiflora*	Kaempferol 3-neohesperidoside	Rat soleus muscle	Enhances glycogen synthesis	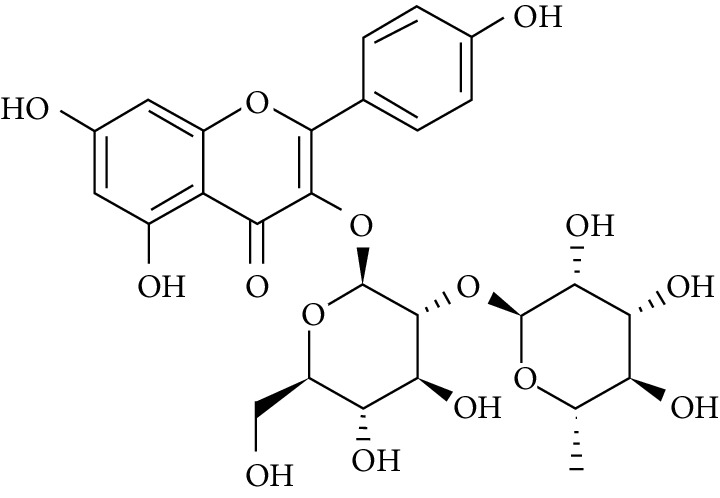	[366]

11	*Maclura pomifera*, *Maclura tinctoria*, and *Psidium guajava*	Morin	HepG2	Enhances the phosphorylation of the insulin receptor, Akt, and FOXO1, hinders gluconeogenesis, and enhances glycogen synthesis	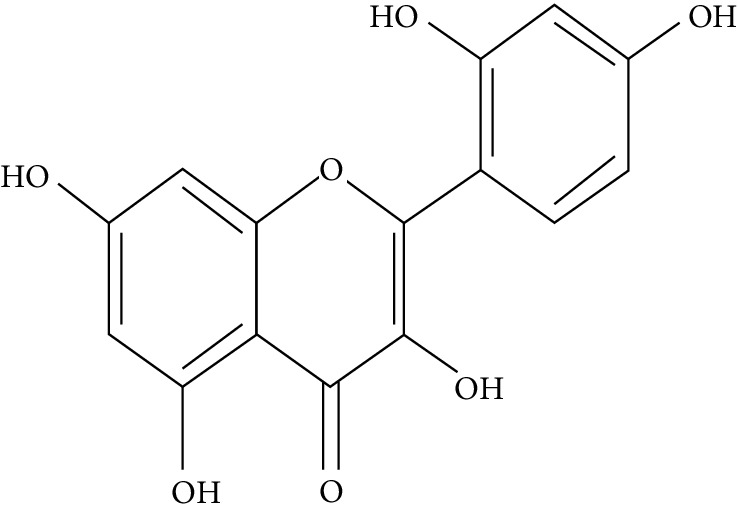	[367]

12	Black ginger (*Kaempferia parviflora* Wall.)	Pentamethyl quercetin	3T3-L1 cell	Elevation of GLUT 4 and PPAR levels in mRNA	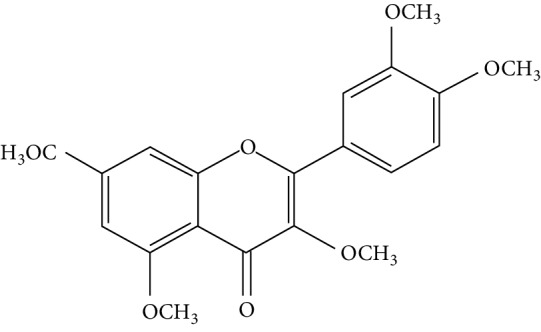	[368]

13	*Curcuma domestica valeton*, *Cuscuta reflexa*, and *Daucus carota*	Quercetin	3T3-L1 adipocytes	Enhanced glucose uptake and mitigated hyperglycemia and PPAR*γ* agonist activity	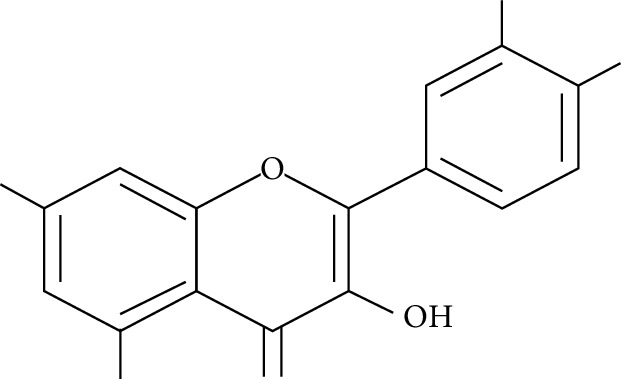	[365]

14	*Solanum crinitum* Lam	Tetramethylkaempferol	3T3-L1 cell	Elevation of GLUT 4 and PPAR levels in mRNA	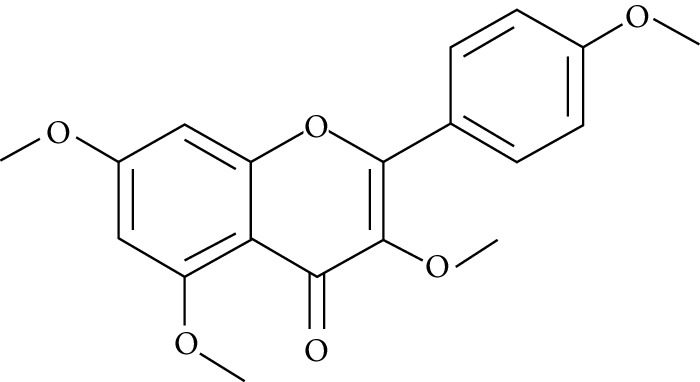	[368]

15	*Tetracera scandens*	Genistein	INS-1 rat insulinoma cells	Stimulated insulin secretion via activation of Ca^2+^/CaMK II	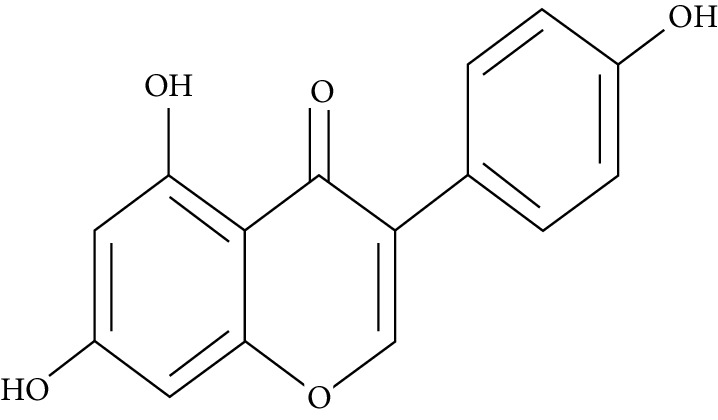	[369]
